# Metformin Enhances the Chemosensitivity of Gastric Cancer to Cisplatin by Downregulating Nrf2 Level

**DOI:** 10.1155/ancp/5714423

**Published:** 2025-04-15

**Authors:** Guihua Duan, Min Qi, Linting Xun, Ying An, Zan Zuo, Yusi Luo, Zhengji Song

**Affiliations:** ^1^Department of Gastroenterology, The First People's Hospital of Yunnan Province, The Affiliated Hospital of Kunming University of Science and Technology, Kunming, Yunnan, China; ^2^Department of Radiology, The Third People's Hospital of Kunming City, The Sixth Affiliated Hospital of Dali University, Kunming, Yunnan, China

**Keywords:** AMPK, cisplatin resistance, gastric cancer, metformin, Nrf2, p53

## Abstract

Cisplatin-based chemotherapy resistance is a common issue for cancer clinical efficacy. Metformin is being studied for its possible anticancer effect. The present study aimed to investigate whether metformin affects the chemosensitivity of gastric cancer to cisplatin and reveal the molecular mechanism. In this study, the effects of combination therapy with metformin and cisplatin on cell viability, cell apoptosis, malondialdehyde, superoxide dismutase, reactive oxygen species level, glucose uptake, lactate production, protein level, and xenograft tumor formation were analyzed in gastric cancer cells. Immunohistochemical staining was performed to detect Ki67 expression in matched tumor samples. The results showed that NCI-N87 and SNU-16 cells were most resistant and sensitive to cisplatin, respectively. Metformin treatment increased the cisplatin sensitivity of gastric cancer by inhibiting cell viability and metabolic reprogramming and promoting cell apoptosis and oxidative stress. Furthermore, overexpression of nuclear factor erythroid 2-related factor 2 (Nrf2) reversed the effects of metformin in the cisplatin sensitivity of gastric cancer by inhibiting cell viability and metabolic reprogramming and promoting cell apoptosis and oxidative stress. Metformin activated p53 and AMPK pathways in cisplatin-induced NCI-N87 cells, which were reversed by upregulating Nrf2. BAY-3827 (AMPK inhibitor) or p-nitro-Pifithrin-α (p53 inhibitor) treatments also reversed the effects of metformin increased the cisplatin sensitivity of gastric cancer by inhibiting cell viability and metabolic reprogramming and promoting cell apoptosis and oxidative stress. These results suggest that metformin significantly increases chemosensitivity of gastric cancer to cisplatin by inhibiting Nrf2 expression and metabolic reprogramming and activating oxidative stress and the pathway of p53 and AMPK.

## 1. Introduction

Gastric cancer is the fifth most frequently diagnosed cancer in the world in 2020 and the third leading cause of cancer deaths worldwide [1]. Many influences contribute to the disease, such as diet, life style, genetic talent, family history, treatments and medical conditions, infections (*Helicobacter pylori* and human papilloma virus), and demographic characteristics [2]. The surgical resection with adequate lymphadenectomy is the only potentially curative treatment approach for patients with gastric cancer [1]. Due to late diagnosis and poor response to existing treatments, most gastric cancer patients typically have poor prognosis [3]. Cisplatin-based chemotherapy remains the primary treatment for advanced gastric cancer [4, 5], but developing chemotherapy resistance is the most vital challenge for clinical efficacy [6]. Therefore, the discovery of a novel and effective treatment strategy to enhance cisplatin sensitivity is vital for improving the prognosis and survival of advanced gastric cancer patients.

Tumor cells increase energy demands required for rapid proliferation, invasion, and metastasis through metabolic reprogramming [7]. Metabolic reprogramming is regulated by various factors, including oncogenes, tumor–host cell interactions, growth factors, and tumor suppressor genes [8]. In addition to regulating the development and progression of tumors, metabolic reprogramming plays essential roles in oxidative stress, apoptosis, proliferation, and related signaling pathway [9–11]. These factors are strongly associated with the chemotherapy resistance of cisplatin [12–14], suggesting that metabolic reprogramming may be an endogenous metabolic process for enhancing cisplatin resistance. Metabolic reprogramming regulates cisplatin resistance through oxidative stress, apoptosis, proliferation, and the signaling pathway of p53 and adenosine 5′-monophosphate-activated protein kinase (AMPK) [15–19]. However, few studies have revealed that metabolic reprogramming is concerned about the cisplatin resistance of gastric cancer [16, 20].

Type 2 diabetes-associated metabolic traits such as hyperglycemia, hyperinsulinemia, inflammation, oxidative stress, and obesity are well-known risk factors for cancer [21]. Metformin is a widely used biguanide drug for its safety and low cost. In addition to treating type 2 diabetes, metformin has also been discovered to be used to treat other diseases, such as pulmonary fibrosis, cancers, obesity, and liver diseases [20]. Metformin therapy can decrease the risk of gastric cancer and breast cancer among patients with type 2 diabetes [22, 23]. A systematic meta-analysis reported that metformin is a useful adjuvant agent in colorectal and prostate cancer radiotherapy [24]. However, current evidence from phase II clinical trials does not support that additional use of metformin could improve the survival outcome in women with breast cancer [25]. Notably, it has been reported that metformin prevents resistance to cisplatin by regulating oxidative stress, apoptosis, metabolic reprogramming, and the signaling pathway of serine/threonine kinase mechanistic target of rapamycin (mTOR), AMPK, and insulin-like growth factor (IGF) in multiple cancers, such as triple-negative breast cancer [26], lung cancer [15, 27], gastric cancer [28], and human nasopharyngeal carcinoma [29]. Considering the critical roles of oxidative stress, apoptosis, and metabolic reprogramming in causing chemotherapy resistance, metformin may regulate these processes and play a vital role in the cisplatin resistance of gastric cancer. However, metformin's role in gastric cancer is not completely understood. Thus, to investigate the effects and potential mechanisms of metformin in the cisplatin resistance of gastric cancer is vital.

## 2. Materials and Methods

### 2.1. Cell Lines and Culture

The KATOIII (BNCC339570), Hs-746T (BNCC100957), NCI-N87 (BNCC341304), and SNU-16 (BNCC359595) cell lines were purchased from Beijing Beina Chuanglian Biotechnology Research Institute (Beijing, China). Hs-746T and KATOIII cells were cultured in Dulbecco's minimal essential medium (DMEM-H, Gibco) and Iscove's modified DMEM-H (IMDM, Gibco) with 10% fetal bovine serum (FBS, Sangon Biotech), respectively. NCI-N87 and SNU-16 cells were cultured in Roswell Park Memorial Institute 1640 (RPMI-1640, Gibco) with 10% FBS. All the cells were grown at 37°C in an atmosphere containing 5% CO_2_.

### 2.2. Cells Treatment

Gastric cancer cells were cultured in 96-well plates (5 × 10^3^ cells/well) until the cell attachment. Then cells were treated with different concentration (0, 5, 10, 50, 100, 500, and 1000 μM) of cisplatin for 3 days. Metformin (MT, D150959-5G, Sigma-Aldrich) [30], BAY-3827 (BAY, an AMPK inhibitor) [31], or p-nitro-Pifithrin-*α* (pnPa, a p53 inhibitor) [32] with 100 μM, 1.4 nM, and 10 μM, respectively, was added to the cells after cisplatin treatment. Cell Proliferation and Cytotoxicity Assay Kit (CCK-8, Solarbio, Beijing, China) was added and incubated for 1 h. Finally, the optical density was measured at 450 nm using a microplate reader (BioTek Instruments Inc). GraphPad Prism 7.00 (GraphPad Software, Inc.) was used to determine IC_50_.

### 2.3. Flow Cytometry

The Annexin V-FITC/PI Cell Apoptosis Detection Kit (Solarbio, Beijing, China) was used to determine cell apoptosis. In brief, 2 × 10^5^ cells were gathered and then 10 μL Annexin V-FITC/PI was added to each well and cultured in the dark for 15 min under room temperature. To determine reactive oxygen species (ROS), the ROS-ID Total ROS detection kit (Enzo Life Sciences, New York, USA) was used according to the manufacturer's instructions. In brief, 2′, 7′-dichlorofluorescein diacetate (DCFH-DA) was added and incubated at 37°C for 25 min and with 3 phosphate-buffered saline (PBS) wash. FACS Verse flow cytometer (Becton Dickinson Biosciences, NJ, USA) and FlowJo software (version 10; Treestar, OR, USA) were used to analysis the apoptosis rate and ROS level.

### 2.4. Glucose Uptake and Lactate Production Assays

Cells were seeded in 6-well plate and incubated with each treatment at 37°C for 12 h. Media on cells was replaced with DMEM-free phenol high glucose media (4 mg/L) and incubated for 1 h at 37°C. After incubation, the glucose uptake of media from each well was measured by Glucose Assay kit (Jiancheng Bioengineering Institute, Nanjing, China) according to the manufacturer's instructions. Media on cells was replaced with phenol red-free Roswell Park Memorial Institute (RPMI) medium without FBS and incubated for 1 h at 37°C. After incubation, lactate was measured by Lactic Acid assay kit (Jiancheng Bioengineering Institute, Nanjing, China) according to the manufacturer's instructions. Cell numbers were counted by a microscope, and the glucose and lactate content were normalized by cell number.

### 2.5. Malondialdehyde Content and Superoxide Dismutase Activity Assays

Cells were seeded in 6-well plate and incubated with each treatment at 37°C for 24 h. After treatment for 8 days, the tumors from each group of mice were excised and malondialdehyde (MDA) content and superoxide dismutase (SOD) activity were measured. The cells and tissues were lysed by the MDA lysis solution in a MDA assay kit (Jiancheng Bioengineering Institute, Nanjing, China). The cells and tissues lysate were centrifuged at 13,000x*g* for 10 min, and the supernatant was collected. The MDA content was determined in the supernatant following the manufacturer's instructions. The cells and tissues were lysed by the SOD buffer in a SOD assay kit (Jiancheng Bioengineering Institute, Nanjing, China). The SOD activity was determined in the supernatant following the manufacturer's instructions.

### 2.6. Nrf2 Overexpression Assay

NCI-N87 cells (3 × 10^5^) were seed in six-well plates and transfected with erythroid 2-related factor 2 (Nrf2) overexpression (oe-Nrf2, 100 nM) plasmid using Lipofectamine 2000 (Invitrogen; Thermo Fisher Scientific, Inc.) following the manufacturer's instructions. After transfection for 48 h, transfection efficiency was detected by Western blot.

### 2.7. *In Vivo* Subcutaneous Tumor Growth Xenograft Model

Six–eight weeks (20–25 g) male C57BL/6 mice were obtained from the Animal Experiment Center of Kunming Medical University (Certificate no. SCXKK2020-0004). All animal experimental protocols were approved by the Institutional Animal Care and Use Committee at Kunming University of Science and Technology. Fifty-five mice were randomly divided in to the following groups: (1) Vehicle: NCI-N87 or SNU-16 cells were injected into each mouse; (2): CDDP: Vehicle group mice receiving 2 mg/kg cisplatin treatment (tail injection); (3) CDDP + MT: CDDP group mice receiving metformin treatment (diluted in drinking water, 100 mg/kg/day); (4) CDDP + MT + BAY: CDDP + MT group mice receiving 10 mg/kg/day BAY treatment (tail injection); and (5) CDDP + MT + pnPa: CDDP + MT group mice receiving 200 mg/kg/day pnPa treatment (tail injection); (6) CDDP + MT + BAY + pnPa: CDDP + MT + BAY group mice receiving 200 mg/kg/day pnPa treatment (tail injection). A total of 4 × 10^6^ cells in each group were dispersed in 2 mL physiological saline and injected into each group of mice. The tumor volume in the mice was measured every 2 days after injection. The tumor volume (V) was calculated as length × width × width × 0.52. After 8 days, mice were killed by overdose of pentobarbital sodium (120 mg/kg, I.P.), and the tumors were excised and weighed.

### 2.8. Western Blot

Cells and tissues were treated as described in the corresponding section of results, and total proteins were extracted from gastric cancer cells or tissues and concentration was measured by bicinchoninic acid (BCA) kit (Beyotime Institute of Biotechnology). For electrophoresis, 20 µg of protein samples were loaded in each lane and were separated using 10% sodium dodecyl sulfate-polyacrylamide gel electrophoresis (SDS-PAGE) system. Briefly, the membrane was incubated with a primary antibodies: Nrf2 (1:1000; no. ab137550; Abcam, UK), AMPK (1:1000; no. ab271188; Abcam), P-AMPK (1:1000; no. ab133448; Abcam), and p53 (1:1000; no. ab32389; Abcam) overnight at 4°C, with 3 Tris Buffered Saline (TBS) wash after each incubation. The blots were visualized using enhanced chemiluminescence reagent (Millipore, Billerica, MA, USA) with β-Actin (1:5000; no. ab8226; Abcam) as an internal loading control.

### 2.9. Immunohistochemistry

The tumor tissue from xenograft models was fixed with 4% paraformaldehyde solution at 4°C overnight. Sections were stained overnight with antibodies against Ki-67 (1:1000; no. ab15580; Abcam) at 4°C, and they were performed using anti-mouse secondary antibody at 37°C for 30 min. Then, according to the manufacturer's instructions (SK-4100; Vector laboratories), the avidin–biotin peroxidase complex was used, followed by colorimetric detection using diaminobenzidine (DAB). Finally, hematoxylin was used to counterstain the sections.

### 2.10. Bioinformatics Analysis

The mRNA expression level of Nrf2 in different normal human organs, and tumor tissues was reviewed by using gene expression profiling interactive analysis (GEPIA: http://gepia.cancer-pku.cn/) online software [33]. TNM stage and survival and the association between NFE2L2 expression were studied by using GEPIA.

### 2.11. Statistical Analysis

Results are expressed as the mean ± standard deviation (SD). GraphPad Prism 7.0 was used for statistical analyses. Two groups were compared with Student's *t*-test, the differences between the control group and experimental groups were determined by one-way analysis of variance following the Tukey–Kramer post hoc analysis. *p*-value < 0.05 was considered significant.

## 3. Results

### 3.1. Determination of Cisplatin Sensitivity in Gastric Cancer Cells

**Figure 1:**
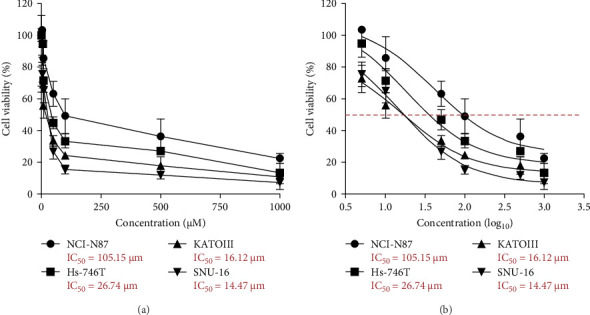
Cisplatin sensitivity was tested in gastric cancer cells. (A) Cell viability was measured with different concentration of cisplatin. (B) IC_50_ values were calculated using GraphPad software.

CCK-8 assay was performed to evaluate the cytotoxicity of cisplatin in four gastric cancer cell lines. As shown in Figure 1, upon cisplatin treatment, the IC_50_ of NCI-N87, Hs-746T, KATOIII, and SNU-16 were 105.15, 26.74, 16.12, and 14.47 μM, respectively. The cytotoxic profile suggested that NCI-N87 and SNU-16 is the gastric cancer cell line which confer most resistant and sensitive, respectively, to cisplatin. Therefore, NCI-N87 and SNU-16 cell lines were selected for further study.

### 3.2. Metformin Sensitizes Gastric Cancer Cells to Cisplatin

**Figure 2:**
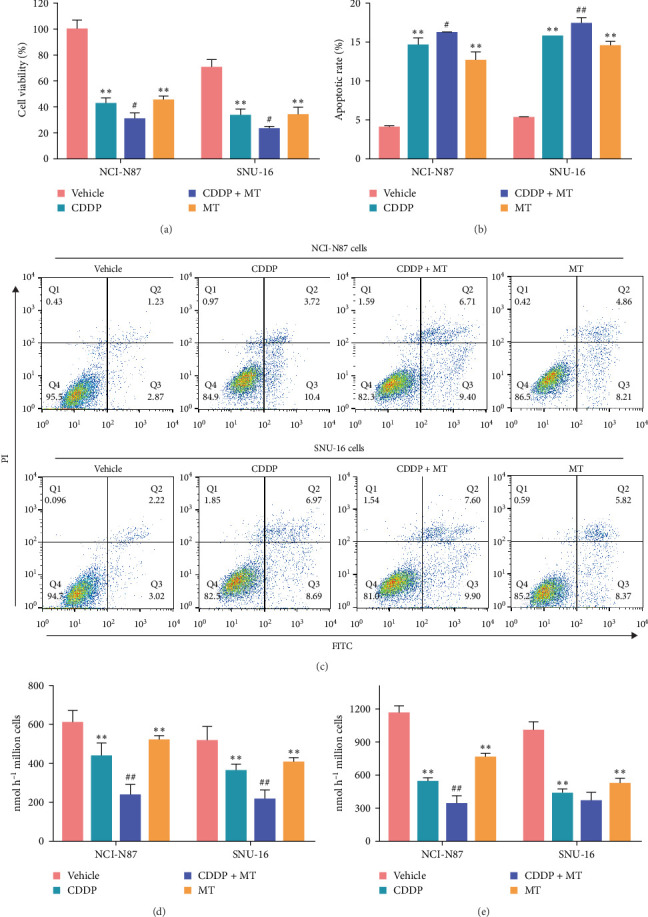
Metformin sensitizes gastric cancer cells to cisplatin. Cell viability (A), cell apoptosis (B, C), glucose uptake (D), and lactate production (E) were measured with different treatments. *⁣*^*∗∗*^*p* < 0.01 vs. Vehicle, ^#^*p* < 0.05 and ^##^*p* < 0.01 vs. CDDP. CDDP, cisplatin; MT, metformin; NCI-N87, NCI-N87 cells; SNU−16, SNU−16 cells.

Metformin prevents resistance to cisplatin by regulating multiple ways in cancer cells [15, 26–29, 34]. We examined the effect of metformin in cisplatin-induced proliferation and apoptosis of gastric cancer cells. As shown in Figure 2A, the cell viability was significantly inhibited after cisplatin and metformin administration in both NCI-N87 and SNU-16 cell lines, and the inhibitor effect of cisplatin was exacerbated with metformin treatment. Furthermore, cisplatin and metformin markedly increased the apoptosis rate of gastric cancer cells, and the effect of cisplatin was significantly aggravated by metformin treatment (Figure 2B). Previous studies identified that metabolic reprogramming did not participate in cisplatin resistance but was also regulated by metformin [15, 35, 36]. Thus, we also measured glucose uptake and lactate production. We observed that cisplatin and metformin significantly inhibited glucose uptake and lactate production of both NCI-N87 and SNU-16 cell lines, and the inhibitor effect of cisplatin was boosted by metformin treatment (Figures 2C,D). These data indicated that metformin increases the sensitivity of gastric cancer cells to cisplatin.

### 3.3. Metformin Sensitizes Gastric Cancer Cells to Cisplatin *In Vivo*

**Figure 3:**
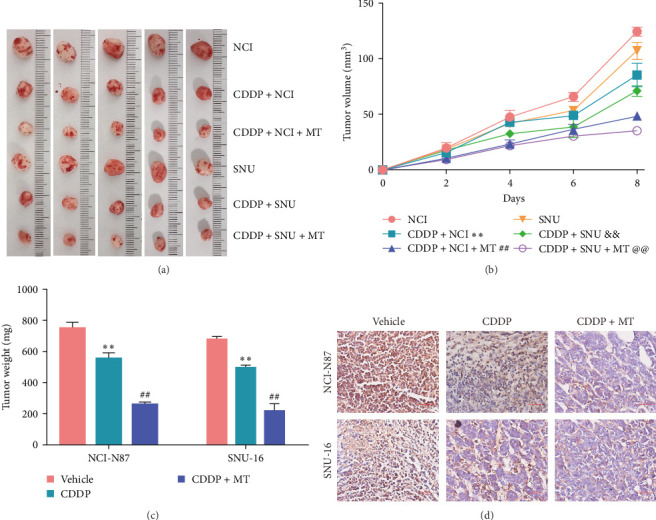
Metformin sensitizes gastric cancer cells to cisplatin *in vivo*. (A) After treatment for 8 days, the tumors from each group of mice were excised and photographed (*n* = 5). (B) Tumor volumes were measured every 2 days (*n* = 5). (C) After treatment for 8 days, tumors were excised and weighed (*n* = 5). (D) Ki−67 immunohistochemistry examinations in the different treatment groups. *⁣*^*∗∗*^*p* < 0.01 vs. NCI-N87 and Vehicle, ^##^*p* < 0.01 vs. CDDP and CDDP + NCI-N87, ^&&^*p* < 0.01 vs. SNU, ^@@^*p* < 0.01 vs. CDDP + SNU. CDDP, cisplatin; MT, metformin; NCI, NCI-N87 cells; SNU, SNU−16 cells.

Since metformin sensitizes gastric cancer cells to cisplatin *in vitro*, we tested whether metformin regulates the sensitivity of gastric cancer cells to cisplatin in a mouse model. The tumor weights and volume were decreased after cisplatin treatment and these effects were aggravated with the application of metformin (Figures 3A–C). As additional markers of proliferation, the Ki-67 expression was also examined. Administration of cisplatin inhibited Ki-67 expression in the tumor tissue of xenograft model, and the effect was aggravated with the application of metformin (Figure 3D). These data suggested that metformin increases the sensitivity of gastric cancer cells to cisplatin *in vivo*.

### 3.4. Metformin Increases Oxidative Stress in Cisplatin-Induced Gastric Cancer

**Figure 4:**
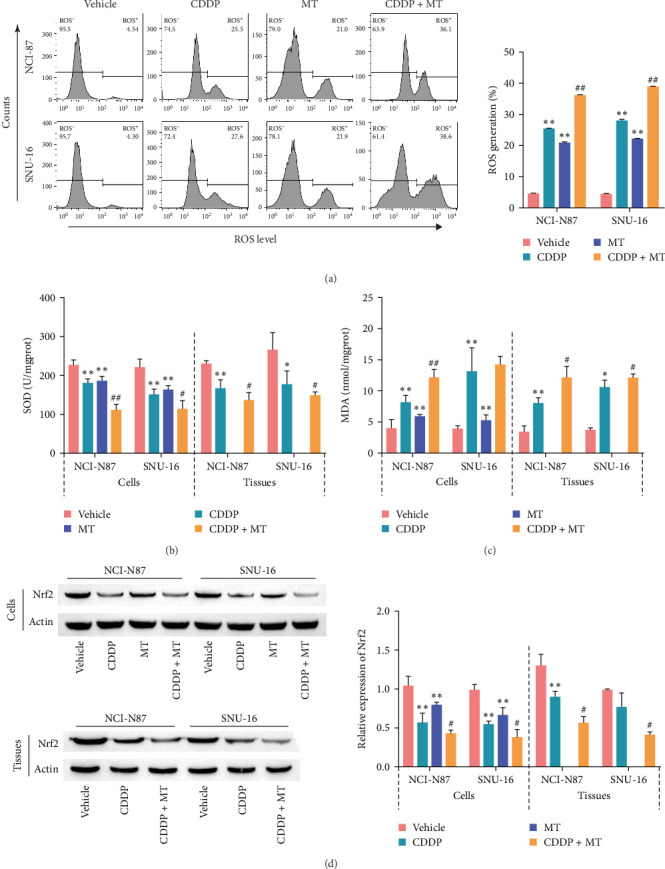
Metformin increases oxidative stress in cisplatin-induced gastric cancer. ROS level (A), SOD activity (B), and MDA content (C) were determined after MT or CDDP treatments in two cell lines. (D) The level of Nrf2 was determined by western blot analysis in cells and tumor tissues. *⁣*^*∗*^*p* < 0.05 and *⁣*^*∗∗*^*p* < 0.01 vs. Vehicle, ^#^*p* < 0.05 and ^##^*p* < 0.01 vs. CDDP. CDDP, cisplatin; MDA, malondialdehyde; MT, metformin; NCI-N87, NCI-N87 cells; SNU−16, SNU−16 cells; Nrf2, nuclear factor erythroid 2-related factor 2; ROS, reactive oxygen species; SOD, superoxide dismutase.

Oxidative stress is closely related to cisplatin resistance in cancer cells [37, 38], we next evaluated the ability of metformin to regulate the oxidative stress level in cisplatin-induced gastric cancer cells. Cisplatin and metformin effectively promoted ROS production of gastric cancer cells, and combining cisplatin and metformin treatment also increased the level of ROS compared with cisplatin-induced cells (Figure 4A). In addition, the activity of SOD (an antioxidative enzyme) was decreased in the cisplatin-treated cells and tissues of gastric cancer, and these effects were raised by metformin treatment (Figure 4B). On the contrary, cisplatin increased the content of MDA (an oxidative stress marker) in the cisplatin-treated cells and tissues of gastric cancer and which was aggravated with metformin administration (Figure 4C). We observed that metformin decreased and increased SOD activity and MDA content, respectively (Figures 4B,C). Furthermore, Nrf2 acted as an inhibitor of oxidative stress and showed a decreased protein level in the cisplatin-induced cells and tissues, and Nrf2 expression was aggravated with combination of cisplatin and metformin treatment (Figure 4D). Metformin treatment also reduced the expression of Nrf2 in gastric cancer cells (Figure 4D). The collective finding indicated that metformin promotes oxidative stress in cisplatin-induced gastric cancer.

### 3.5. Metformin Promotes the Cisplatin Sensitivity of Gastric Cancer Cells by Downregulating Nrf2

Our data found that Nrf2 was downregulated after cisplatin or metformin treatment. Then, we used the bioinformatics website, GEPIA, to investigate whether the Nrf2 protein is involved in the occurrence and development of gastric cancer. The Nrf2 protein gene, *NFE2L2*, expression was not significantly changed in gastric cancer tissues (*n* = 408) compared with normal gastric tissues (*n* = 211) only a few cancer tissues exhibited differential expression (Figure S1A,B). In addition, there was no significant difference in TNM stage, disease-free survival, and overall survival with *NFE2L2* expression (Figure S1C–E). These results suggested that Nrf2 is not involved in the occurrence and development of gastric cancer.

**Figure 5:**
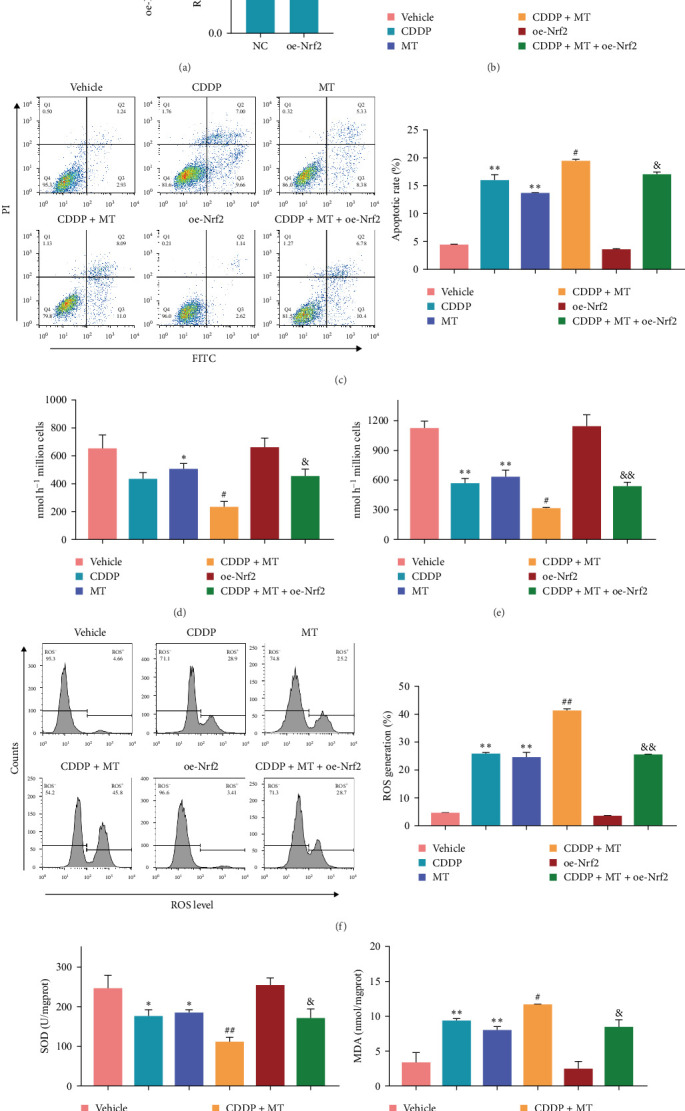
Metformin promotes the cisplatin sensitivity of NCI-N87 cells by down-regulating Nrf2. (A) The level of Nrf2 was determined in the oe-Nrf2 NCI-N87 cells. *⁣*^*∗∗*^*p* < 0.01 vs. NC. Cell viability (B), cell apoptosis (C), glucose uptake (D), lactate production (E), ROS level (F), SOD activity (G), and MDA content (H) were determined in different treatment NCI-N87 cells. *⁣*^*∗*^*p* < 0.05 and *⁣*^*∗∗*^*p* < 0.01 vs. Vehicle, ^#^*p* < 0.05 and ^##^*P* < 0.01 vs. CDDP, ^&^*p* < 0.05 and ^&&^*p* < 0.01 vs. CDDP + MT. CDDP, cisplatin; MDA, malondialdehyde; MT, metformin; oe-Nrf2, over-expression Nrf2; ROS, reactive oxygen species; SOD, superoxide dismutase.

Considering Nrf2 expression was downregulated in cisplatin-induced gastric cancer cells and tissues and metformin-induced gastric cancer cells, then we upregulated Nrf2 protein level in NCI-N87 cells. Western blot results displayed that Nrf2 protein level was significantly increased (Figure 5A). Cell viability was inhibited after metformin and cisplatin treatment in NCI-N87 cells (Figure 5B). Metformin decreased the viability of cisplatin-induced NCI-N87 cells but was reversed with Nrf2 overexpression. On the contrary, cisplatin and metformin elevated the apoptosis rate of NCI-N87 cells (Figure 5C). Metformin increased the apoptosis rate of cisplatin-induced NCI-N87 cells, which was reversed with overexpression of Nrf2 (Figure 5C). Furthermore, the glucose uptake (Figure 5D) and lactate production (Figure 5E) were suppressed by cisplatin and metformin treatment, the inhibitor effect of cisplatin was enhanced by metformin treatment, but the glucose uptake and lactate production were finally boosted by Nrf2 overregulation. In addition, metformin led to the cisplatin-induced NCI-N87 cells ROS level increase (Figure 5F), SOD activity decrease (Figure 5G), and MDA content elevate (Figure 5H), while the effect of metformin treatment were reversed with overexpression of Nrf2. These data showed that metformin promotes the cisplatin sensitivity of gastric cancer cells by downregulating Nrf2.

### 3.6. Metformin Activates p53 and AMPK Pathways in Cisplatin-Induced NCI-N87 Cells by Downregulating Nrf2

**Figure 6:**
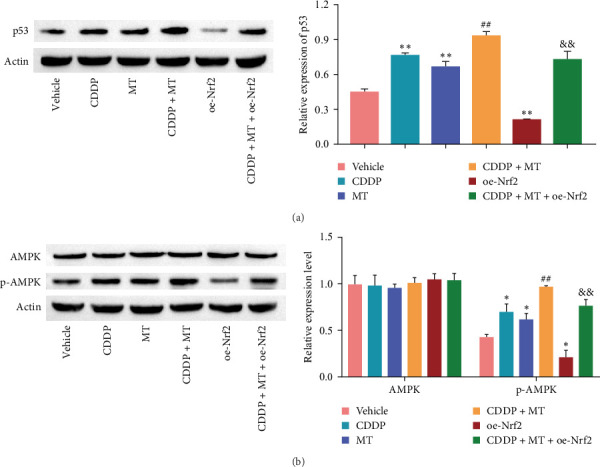
Metformin activates p53 and AMPK pathways in cisplatin-induced NCI-N87 cells by down-regulating Nrf2. The level of p53 (A) and AMPK (B) pathways were measured by western blot in different treatment groups. *⁣*^*∗*^*p* < 0.05 and *⁣*^*∗∗*^*p* < 0.01 vs. Vehicle, ^##^*p* < 0.01 vs. CDDP, ^&&^*p* < 0.01 vs. CDDP + MT. AMPK, adenosine 5′-monophosphate-activated protein kinase; CDDP, cisplatin; MT, metformin; oe-Nrf2, over-expression nuclear factor erythroid 2-related factor 2.

Previous studies have shown that the pathway of p53 and AMPK regulate metabolic reprogramming, cisplatin resistance, oxidative stress, and apoptosis [15–19]. Thus, we detected the activation of p53 and AMPK pathway in NCI-N87 cells. As shown in Figure 6, cisplatin and metformin activated the pathway of p53 (Figure 6A) and AMPK (Figure 6B), and metformin increased the cisplatin-induced p53 and AMPK pathways activation, but the increasing effect finally was reversed with overexpression of Nrf2. These data suggested that metformin activates the pathway of p53 and AMPK in cisplatin-induced NCI-N87 cells by downregulating Nrf2.

### 3.7. Metformin Promotes the Cisplatin Sensitivity of NCI-N87 Cells by Activating p53 and AMPK Pathways

**Figure 7:**
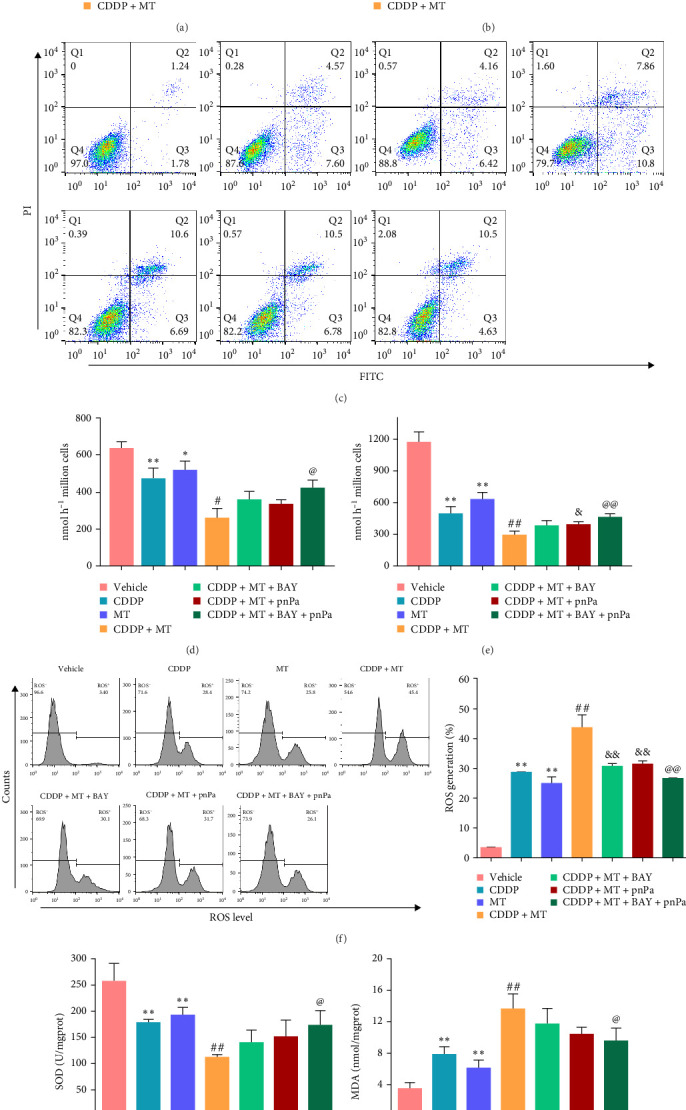
Metformin promotes the cisplatin sensitivity of NCI-N87 cells by activating p53 and AMPK pathways. Cell viability (A), cell apoptosis (B, C), glucose uptake (D), lactate production (E), ROS level (F), SOD activity (G), MDA content (H) were determined in CDDP-induced cells with p53 or AMPK inhibitor. *⁣*^*∗*^*p* < 0.05 and *⁣*^*∗∗*^*p* < 0.01 vs. Vehicle, ^#^*p* < 0.05 and ^##^*p* < 0.01 vs. CDDP, ^&^*p* < 0.05, ^&&^*p* < 0.01, ^@^*p* < 0.05, and ^@@^*p* < 0.01 vs. CDDP + MT. BAY, BAY−3827; CDDP, cisplatin; MDA, malondialdehyde; MT, metformin; pnPa, p-nitro-Pifithrin-*α*; ROS, reactive oxygen species; SOD, superoxide dismutase.

Our finding that metformin can significantly activate the pathway of p53 and AMPK in cisplatin-induced NCI-N87 cells. The inhibitors of pnPa (p53 inhibitor) and BAY (AMPK inhibitor) were used to inhibit p53 and AMPK pathways' activation in the NCI-N87 cells, respectively. The viability of NCI-N87 cells was decreased with cisplatin and metformin treatments, and metformin treatment inhibited the cell viability of cisplatin-induced NCI-N87 cells but was reversed with BAY and pnPa treatments (Figure 7A). Notably, the effect of metformin-induced cell viability decrease was significantly alleviated by combining BAY and pnPa treatment (Figure 7A). Cell apoptosis was significantly increased by cisplatin and metformin, and the inhibitor effect of cisplatin was exacerbated with metformin treatment, which was repressed by BAY or pnPa treatments (Figure 7B). Metformin inhibited glucose uptake (Figure 7C) and lactate production (Figure 7D) in the cisplatin-induced NCI-N87 cells, which was reversed with treatment of BAY or pnPa. ROS level was elevated after metformin treatment in the cisplatin-induced NCI-N87 cells but reduced with BAY or pnPa treatments (Figure 7E). In addition, SOD activity was reduced after metformin treatment in the cisplatin-induced NCI-N87 cells but elevated with BAY or pnPa treatments (Figure 7F). On the contrary, metformin increased MDA content in the cisplatin-induced NCI-N87 cells, which was reversed with treatments of BAY or pnPa (Figure 7G). These data indicated that metformin promotes the cisplatin sensitivity of gastric cancer cells by activating p53 and AMPK pathways.

### 3.8. Metformin Promotes the Cisplatin Sensitivity of Gastric Cancer by Activating p53 and AMPK Pathways *In Vivo*

**Figure 8:**
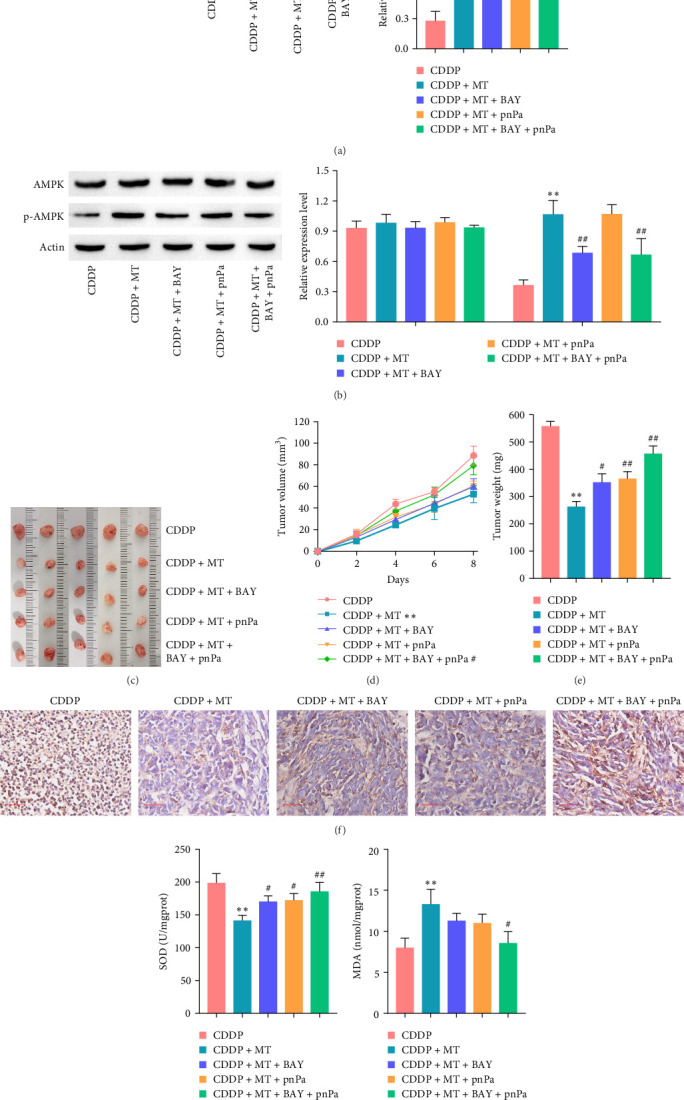
Metformin promotes the cisplatin sensitivity of gastric cancer by activating p53 and AMPK pathways *in vivo*. (A) The p53 (A) and AMPK (B) pathway were measured by western blot in CDDP-induced tumor tissues with p53 or AMPK inhibitor. (C) After treatment for 8 days, the tumors from each group of mice were excised and photographed (*n* = 5). (D) Tumor volumes were measured every 2 days (*n* = 5). (E) After treatment for 8 days, tumors were excised and weighed (*n* = 5). (F) Ki−67 immunohistochemistry examinations in the different treatment groups. After treatment for 8 days, the SOD activity (G) and MDA content (H) were measured in the tumor tissues from each group. *⁣*^*∗∗*^*p* < 0.01 vs. CDDP, ^#^*p* < 0.05 and ^##^*p* < 0.01 vs. CDDP + MT. BAY, BAY-3827; CDDP, cisplatin; MDA, malondialdehyde; MT, metformin; pnPa, p-nitro-Pifithrin-*α*; SOD, superoxide dismutase.

We subsequently validated the molecular mechanism of metformin on cisplatin sensitivity of gastric cancer *in vivo*. As shown in Figures 8A,B, the level of p53 and p-AMPK was elevated after metformin treatment in the cisplatin-induced tumor tissues, which was reversed with pnPa and BAY, respectively. In addition, p53 and AMPK pathways were inhibited with pnPa and BAY treatment in the cisplatin-induced tumor tissues. The tumor weights and volumes were decreased after metformin treatment in the cisplatin-induced tumor tissues, and these effects were reversed with applications of BAY or pnPa (Figures 8C–E). The expression of Ki-67 was inhibited in the cisplatin-induced tumor tissues of xenograft model, and the effect was reversed with treatment of BAY or pnPa (Figure 8F). Furthermore, SOD activity (Figure 8G) and MDA content (Figure 8H) were decreased and increased, respectively, with metformin treatment in the cisplatin-induced tumor tissues, and these effects were reversed with treatments of BAY or pnPa. All together, our data show that metformin promotes the cisplatin sensitivity of gastric cancer by activating p53 and AMPK pathways *in vivo*.

## 4. Discussion

Gastric cancer is profoundly resistant to cisplatin therapy. Novel and effective therapeutic approaches are urgently needed. Numerous studies have shown that metformin is a novel drug for the resistance of cisplatin [26, 34, 39]. This study provided the mechanism of the inhibitor effects of metformin on cisplatin resistance in gastric cancer. We found that it significantly increased the cisplatin sensitivity of gastric cancer, accompanied with elevated cell apoptosis and oxidative stress and inhibited cell proliferation, suggesting that metformin treatment may be a novel option for use in conjunction with cisplatin. Metformin is being studied for its possible antiaging, anticancer, and neuroprotective effects. In addition to regulating these processes, metformin plays important roles in antioxidative stress [40, 41]. However, the oxidative stress of gastric cancer cells and tissues with cisplatin treatment is significantly increased after metformin [40, 41]. Interestingly, the oxidative stress of cancer cells is activated after metformin treatment [42–44], suggesting that metformin elevates oxidative stress in the cancer cells.

Metabolic reprogramming refers to the ability of cancer cells to alter their metabolism in order to support the increased energy request due to continuous growth, rapid proliferation, and other characteristics typical of neoplastic cells [36, 45, 46]. In our study, metformin significantly inhibited glucose uptake and lactate production in gastric cancer cells with cisplatin treatment. Metabolic reprogramming plays a vital role in cisplatin resistance [36, 45, 46]. We observed that metformin increased ROS and MAD levels and decreased SOD production, while glucose uptake and lactate production were inhibited. In a recent report, cisplatin resistance involves metabolic reprogramming through ROS and peroxisome proliferator-activated receptor gamma coactivator-1 alpha (PGC-1*α*) in non-small cell lung cancer [15], suggesting that metformin promotes the cisplatin sensitivity of gastric cancer by elevating oxidative stress and inhibiting metabolic reprogramming.

Nrf2 is a transcription factor to regulate the cellular defense against toxic and oxidative stress [47]. Our studies further showed that Nrf2 was involved in the cisplatin sensitivity of gastric cancer, which was reduced with metformin treatment. Notably, Nrf2 activation renders cells resistant to chemical carcinogens and inflammatory challenges [47]. In our study, we showed that overexpression of Nrf2 reduced gastric cancer cell apoptosis, oxidative stress, and increased cell proliferation. Nrf2 has been shown to contribute to the interplay between redox homeostasis and metabolic alternation within cancer cells [48]. Previous studies have shown that metformin reduces Nrf2 level in non-small cell lung cancer cells [49] and hepatocellular carcinoma cells [50] by increasing the ubiquitination and proteasomal degradation of Nrf2. In our experiments, metformin also reduced Nrf2 expression in both gastric cancer cell lines. In addition, metformin reduces Nrf2 level via accelerating ubiquitination-mediated degradation in non-small cell lung cancer [51]. However, we did not detect the effect of metformin on Nrf2 expression in tumors, which was a limitation of our results. Interestingly, metformin increases the radiosensitivity and chemosensitivity of non-small cell lung cancer by destabilizing Nrf2 [49, 51]. These results indicate that metformin may function as a synergistic agent for sensitivity of cisplatin use.

In our study, metformin activated AMPK and p53 signaling pathways in the cisplatin-induced gastric cancer cells and tissues, while were reversed with overexpression of Nrf2. AMPK pathway is strongly associated with the metabolic reprogramming of cancer [52–54], which is orchestrated with oxidative stress [11]. Queiroz et al. [43] reported that metformin mediates apoptosis and cell cycle arrest by oxidative stress and AMPK pathway in breast cancer cells (MCF-7). The level of p53 is kept low in unstressed cells due to its polyubiquitination by the E3 ubiquitin ligase murine double minute 2 (MDM2) [55], which is activated with oxidative stress [56]. ROS is a common subproduct of oxidative energy metabolism and is considered to be a significant physiological modulator of multiple signaling pathways, including p53 and AMPK pathways [11, 57]. In our study, the level of oxidative stress was increased with cisplatin or metformin treatment, implying that metformin may activate p53 and AMPK pathways by elevating oxidative stress in gastric cancer cells and tissues. Notably, metformin activated p53 and AMPK pathways in cisplatin-induced NCI-N87 cells and tumors, which were reversed with AMPK or p53 pathways inhibitor treatments or upregulation Nrf2. Combined with the function of Nrf2, we speculate that metformin may activate p53 and AMPK pathways by inhibiting Nrf2 expression.

## 5. Conclusion

This study demonstrates that metformin treatment significantly increases the sensitively of cisplatin in gastric cancer cells, the increasing appears to be largely dependent to the inhibition of Nrf2 expression and metabolic reprogramming and the activation of oxidative stress and the pathway of p53 and AMPK. These results provide new insights for reducing the cisplatin resistance of gastric cancer.

## Data Availability

All data are available from the corresponding author with reasonable request.

## Supporting Information

Supporting Information Figure S1: *NFE2L2* is not involved in the occurrence and development of gastric cancer (PDF).

## References

[1] Mukkamalla S., Recio-Boiles A., Babiker H. (2023). *Gastric Cancer*.

[2] Yusefi A., Lankarani K. Bagheri, Bastani P., Radinmanesh M., Kavosi Z. (2018). Risk Factors for Gastric Cancer: A Systematic Review. *Asian Pacific Journal of Cancer Prevention*.

[3] Cristescu R., Lee J., Nebozhyn M. (2015). Molecular Analysis of Gastric Cancer Identifies Subtypes Associated With Distinct Clinical Outcomes. *Nature Medicine*.

[4] Ajani J. A., Abramov M., Bondarenko I. (2017). A Phase III Trial Comparing Oral S-1/Cisplatin and Intravenous 5-Fluorouracil/Cisplatin in Patients With Untreated Diffuse Gastric Cancer. *Annals of Oncology*.

[5] Kang Y., Chin K., Chung H. (2020). S-1 Plus Leucovorin and Oxaliplatin Versus S-1 Plus Cisplatin as First-Line Therapy in Patients With Advanced Gastric Cancer (SOLAR): A Randomised, Open-Label, Phase 3 Trial. *The Lancet Oncology*.

[6] Zhai J., Shen J., Xie G. (2019). Fibroblasts-Derived IL-8 Mediates Resistance to Cisplatin in Human Gastric Cancer. *Cancer Letters*.

[7] Raggi C., Taddei M. L., Rae C., Braconi C., Marra F. (2022). Metabolic Reprogramming in Cholangiocarcinoma. *Journal of Hepatology*.

[8] Nong S., Han X., Xiang Y. (2023). Metabolic Reprogramming in Cancer. *Mechanisms and Therapeutics*.

[9] Avolio R., Matassa D., Criscuolo D., Landriscina M., Esposito F. (2020). Modulation of Mitochondrial Metabolic Reprogramming and Oxidative Stress to Overcome Chemoresistance in Cancer. *Biomolecules*.

[10] Schiliro C., Firestein B. (2021). Mechanisms of Metabolic Reprogramming in Cancer Cells Supporting Enhanced Growth and Proliferation. *Cells*.

[11] Zhao Y., Hu X., Liu Y. (2017). ROS Signaling Under Metabolic Stress: Cross-Talk Between AMPK and AKT Pathway. *Molecular Cancer*.

[12] Wang S., Chen Z., Zhu S. (2020). PRDX2 Protects Against Oxidative Stress Induced by H Pylori and Promotes Resistance to Cisplatin in Gastric Cancer. *Redox Biology*.

[13] Galluzzi L., Senovilla L., Vitale I. (2012). Molecular Mechanisms of Cisplatin Resistance. *Oncogene*.

[14] Wang D., Zhao C., Xu F. (2021). Cisplatin-Resistant NSCLC Cells Induced by Hypoxia Transmit Resistance to Sensitive Cells Through Exosomal PKM2. *Theranostics*.

[15] Cruz-Bermúdez A., Laza-Briviesca R., Vicente-Blanco R. (2019). Cisplatin Resistance Involves a Metabolic Reprogramming Through ROS and PGC-1*A* in NSCLC Which Can Be Overcome by OXPHOS Inhibition. *Free Radical Biology and Medicine*.

[16] Dai Y., Li F., Jiao Y. (2021). Mortalin/Glucose-Regulated Protein 75 Promotes the Cisplatin-Resistance of Gastric Cancer via Regulating Anti-Oxidation/Apoptosis and Metabolic Reprogramming. *Cell Death Discovery*.

[17] Tan Y., Li J. (2022). Metabolic Reprogramming from Glycolysis to Fatty Acid Uptake and Beta-Oxidation in Platinum-Resistant Cancer Cells. *Nature Communications*.

[18] Han C., Patten D., Lee S. (2019). Tsang, p53 Promotes Chemoresponsiveness by Regulating Hexokinase II Gene Transcription and Metabolic Reprogramming in Epithelial Ovarian Cancer. *Molecular Carcinogenesis*.

[19] Barisciano G., Colangelo T., Rosato V. (2020). MiR-27a Is a Master Regulator of Metabolic Reprogramming and Chemoresistance in Colorectal Cancer. *British Journal of Cancer*.

[20] Lv Z., Guo Y. (2020). Metformin and Its Benefits for Various Diseases. *Frontiers in Endocrinology*.

[21] Mirmiran P., Bahadoran Z., Ghasemi A., Hosseinpanah F. (2019). Type 2 Diabetes and Cancer: An Overview of Epidemiological Evidence and Potential Mechanisms. *Critical Reviews in Oncogenesis*.

[22] Zhou X., Xue W., Ding X. (2017). Association Between Metformin and the Risk of Gastric Cancer in Patients With Type 2 Diabetes Mellitus: A Meta-Analysis of Cohort Studies. *Oncotarget*.

[23] Col N. F., Ochs L., Springmann V., Aragaki A. K., Chlebowski R. T. (2012). Metformin and Breast Cancer Risk: A Meta-Analysis and Critical Literature Review. *Breast Cancer Research and Treatment*.

[24] Coyle C., Cafferty F. H., Vale C., Langley R. E. (2016). Metformin as an Adjuvant Treatment for Cancer: A Systematic Review and Meta-Analysis. *Annals of Oncology*.

[25] Wang Q., Ma X., Long J., Du X., Pan B., Mao H. (2022). Metformin and Survival of Women With Breast Cancer: A Meta-Analysis of Randomized Controlled Trials. *Journal of Clinical Pharmacy and Therapeutics*.

[26] Lee J., Kang M., Byun W. (2019). Metformin Overcomes Resistance to Cisplatin in Triple-Negative Breast Cancer (TNBC) Cells by Targeting RAD51. *Breast Cancer Research*.

[27] Morelli A., Jr T., Pavan I. (2021). Metformin Impairs Cisplatin Resistance Effects in A549 Lung Cancer Cells Through Mtor Signaling and Other Metabolic Pathways. *International Journal of Oncology*.

[28] Xiao Y., Xiao J., Wang X. (2022). Metformin-Induced AMPK Activation Promotes Cisplatin Resistance Through PINK1/Parkin Dependent Mitophagy in Gastric Cancer. *Frontiers in Oncology*.

[29] Sun Y., Chen X., Zhou Y. (2020). Metformin Reverses the Drug Resistance of Cisplatin in Irradiated CNE-1 Human Nasopharyngeal Carcinoma Cells Through PECAM-1 Mediated MRPs Down-Regulation. *International Journal of Medical Sciences*.

[30] Lu H., Han X., Ren J., Ren K., Li Z., Zhang Q. (2021). Metformin Attenuates Synergic Effect of Diabetes Mellitus and, *Helicobacter pylori*, Infection on Gastric Cancer Cells Proliferation by Suppressing PTEN Expression. *Journal of Cellular and Molecular Medicine*.

[31] Lemos C., Schulze V., Baumgart S. (2021). The Potent AMPK Inhibitor BAY-3827 Shows Strong Efficacy in Androgen-Dependent Prostate Cancer Models. *Cellular Oncology*.

[32] Shimizu H., Yisireyili M., Nishijima F., Niwa T. (2013). Indoxyl Sulfate Enhances p53-TGF-*Β*1-Smad3 Pathway in Proximal Tubular Cells. *American Journal of Nephrology*.

[33] Tang Z., Li C., Kang B., Gao G., Li C., Zhang Z. (2017). GEPIA: A Web Server for Cancer and Normal Gene Expression Profiling and Interactive Analyses. *Nucleic Acids Research*.

[34] Moro M., Caiola E., Ganzinelli M. (2018). Metformin Enhances Cisplatin-Induced Apoptosis and Prevents Resistance to Cisplatin in Co-Mutated KRAS/LKB1 NSCLC. *Journal of Thoracic Oncology*.

[35] Xiao N., Wang J., Wang T. (2022). Metformin Abrogates Pathological TNF-*A*-Producing B Cells Through Mtor-Dependent Metabolic Reprogramming in Polycystic Ovary Syndrome. *ELife*.

[36] Cazzaniga M., Bonanni B. (2015). Relationship Between Metabolic Reprogramming and Mitochondrial Activity in Cancer Cells. Understanding the Anticancer Effect of Metformin and Its Clinical Implications. *Anticancer Research*.

[37] Kuo C., Babuharisankar A., Lin Y. (2022). Mitochondrial Oxidative Stress in the Tumor Microenvironment and Cancer Immunoescape. *Journal of Biomedical Science*.

[38] Romani A. (2022). Cisplatin in Cancer Treatment. *Biochemical Pharmacology*.

[39] Xie Y., Peng Z., Shi M., Ji M., Guo H., Shi H. (2014). Metformin Combined With P38 MAPK Inhibitor Improves Cisplatin Sensitivity in Cisplatin-Resistant Ovarian Cancer. *Molecular Medicine Reports*.

[40] Ren H., Shao Y., Wu C., Ma X., Lv C., Wang Q. (2020). Metformin Alleviates Oxidative Stress and Enhances Autophagy in Diabetic Kidney Disease Via AMPK/SIRT1-Foxo1 Pathway. *Molecular and Cellular Endocrinology*.

[41] Katila N., Bhurtel S., Park P.-H., Choi D.-Y. (2021). Metformin Attenuates Rotenone-Induced Oxidative Stress and Mitochondrial Damage via the AKT/Nrf2 Pathway. *Neurochemistry International*.

[42] Sena P., Mancini S., Benincasa M., Mariani F., Palumbo C. (2018). Metformin Induces Apoptosis and Alters Cellular Responses to Oxidative Stress in Ht29 Colon Cancer Cells: Preliminary Findings. *International Journal of Molecular Sciences*.

[43] Queiroz E., Puukila S., Eichler R. (2014). Metformin Induces Apoptosis and Cell Cycle Arrest Mediated by Oxidative Stress, AMPK and FOXO3A in MCF-7 Breast Cancer Cells. *PloS One*.

[44] Farrash W., Aslam A., Almaimani R. (2023). Metformin and Thymoquinone Co-Treatment Enhance 5-Fluorouracil Cytotoxicity by Suppressing the PI3K/mTOR/HIF1*A* Pathway and Increasing Oxidative Stress on Colon Cancer Cells. *BioFactors*.

[45] Cioce M., Valerio M., Casadei L. (2014). Metformin-Induced Metabolic Reprogramming of Chemoresistant ALDHbright Breast Cancer Cells. *Oncotarget*.

[46] Mostafavi S., Zalpoor H., Hassan Z. (2022). The Promising Therapeutic Effects of Metformin on Metabolic Reprogramming of Cancer-Associated Fibroblasts in Solid Tumors. *Cellular & Molecular Biology Letters*.

[47] He F., Ru X., Wen T. (2020). NRF2, a Transcription Factor for Stress Response and Beyond. *International Journal of Molecular Sciences*.

[48] Wang Y., Chen J. (2018). Nrf2-Mediated Metabolic Reprogramming in Cancer. *Oxidative Medicine and Cellular Longevity*.

[49] Sun X., Dong M., Gao Y. (2022). Metformin Increases the Radiosensitivity of Non-Small Cell Lung Cancer Cells by Destabilizing NRF2. *Biochemical Pharmacology*.

[50] Tang K., Chen Q., Liu Y., Wang L., Lu W. (2022). Combination of Metformin and Sorafenib Induces Ferroptosis of Hepatocellular Carcinoma Through p62-Keap1-Nrf2 Pathway. *Journal of Cancer*.

[51] Huang S., He T., Yang S. (2020). Metformin Reverses Chemoresistance in Non-Small Cell Lung Cancer via Accelerating Ubiquitination-Mediated Degradation of Nrf2. *Translational Lung Cancer Research*.

[52] Fang J., Chen J., Zheng J. (2023). Fructose Metabolism in Tumor Endothelial Cells Promotes Angiogenesis by Activating AMPK Signaling and Mitochondrial Respiration. *Cancer Research*.

[53] Xiao J., Wang S., Chen L. (2024). 25-Hydroxycholesterol Regulates Lysosome AMP Kinase Activation and Metabolic Reprogramming to Educate Immunosuppressive Macrophages. *Immunity*.

[54] Chen J., Zou L., Lu G. (2022). PFKP Alleviates Glucose Starvation-Induced Metabolic Stress in Lung Cancer Cells Via AMPK-ACC2 Dependent Fatty Acid Oxidation. *Cell Discovery*.

[55] Liebl M., Hofmann T. (2021). The Role of p53 Signaling in Colorectal Cancer. *Cancers*.

[56] Yang H., Xie Y., Yang D., Ren D. (2017). Oxidative Stress-Induced Apoptosis in Granulosa Cells Involves JNK, p53 and Puma. *Oncotarget*.

[57] Reuter S., Gupta S. C., Chaturvedi M. M., Aggarwal B. B. (2010). Oxidative Stress, Inflammation, and Cancer: How Are They Linked?. *Free Radical Biology and Medicine*.

