# Supplementary material for: Metformin Enhances the Chemosensitivity of Gastric Cancer to Cisplatin by Downregulating Nrf2 Level
Source: Anal Cell Pathol (Amst). 2025 Apr 15;2025:5714423. doi: 10.1155/ancp/5714423 (PMC12014253; doi:10.1155/ancp/5714423)
Supplement: Supporting Information — Figure S1: NFE2L2 is not involved in the occurrence and development of gastric cancer (PDF). [file 5714423.f1.pdf]

Supplemental information

The Nrf2 protein gene, *NFE2L2*, down-regulated in the BLCA and OV and was up-regulated in the GBM, LGG, PAAD, and THYM (Figure S1A), but *NFE2L2* expression not significant changed in gastric cancer tissues (n = 408) compared with normal gastric tissues (n = 211) (Figure S1A and S1B). There was no significant difference in TNM stage, disease-free survival, and overall survival with *NFE2L2* expression (Figure S1C-E).

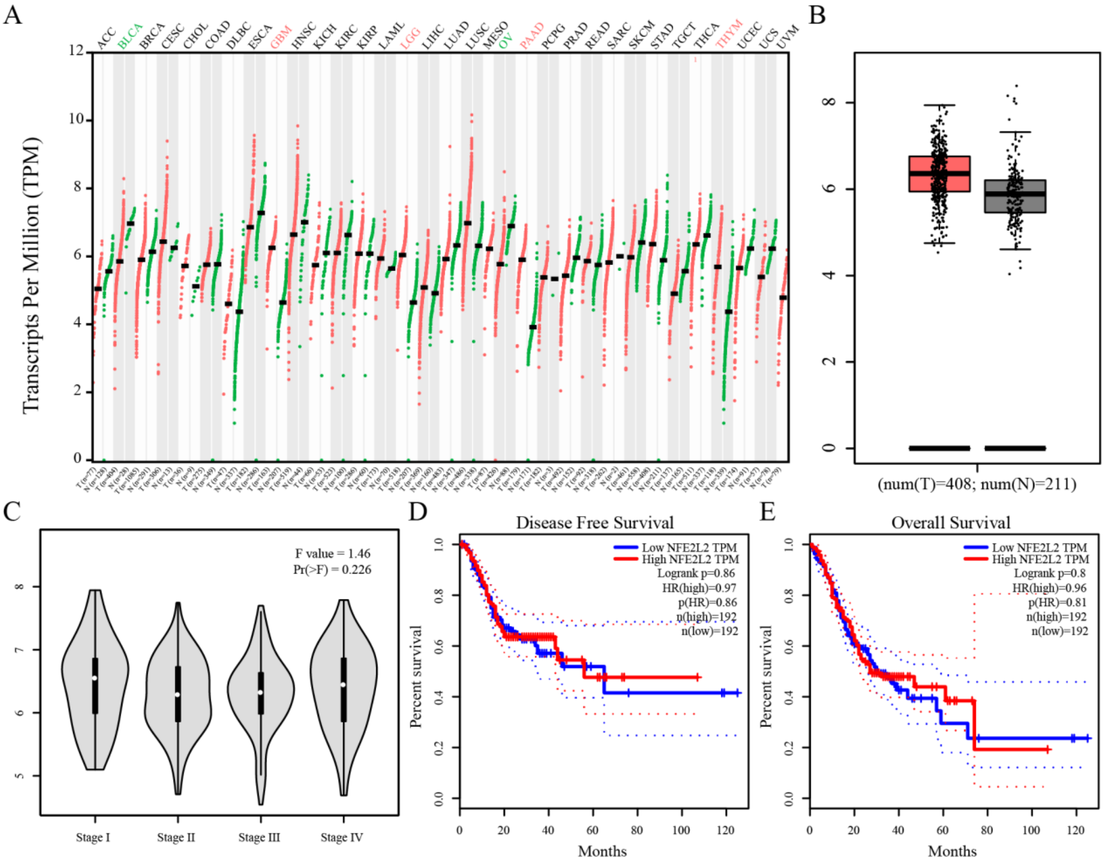

Figure S1. *NFE2L2* is not involved in the occurrence and development of gastric cancer. (A) *NFE2L2* expression in different normal human tissues and cancer tissues. (B) Comparison of *NFE2L2* expression in gastric cancer tissues and in normal liver tissues. (C) *NFE2L2* expression in TNM stage. (D) *NFE2L2* expression in disease free survival. (E) *NFE2L2* expression in overall survival.
